# PyroMEMS as Future Technological Building Blocks for Advanced Microenergetic Systems

**DOI:** 10.3390/mi12020118

**Published:** 2021-01-23

**Authors:** Jean-Laurent Pouchairet, Carole Rossi

**Affiliations:** LAAS-CNRS, University of Toulouse, 7 Avenue du Colonel Roche, 31077 Toulouse, France; jlpoucha@laas.fr

**Keywords:** microenergetics, MEMS, pyroMEMS, IR flare, nanothermite

## Abstract

For the past two decades, many research groups have investigated new methods for reducing the size and cost of safe and arm-fire systems, while also improving their safety and reliability, through batch processing. Simultaneously, micro- and nanotechnology advancements regarding nanothermite materials have enabled the production of a key technological building block: pyrotechnical microsystems (pyroMEMS). This building block simply consists of microscale electric initiators with a thin thermite layer as the ignition charge. This microscale to millimeter-scale addressable pyroMEMS enables the integration of intelligence into centimeter-scale pyrotechnical systems. To illustrate this technological evolution, we hereby present the development of a smart infrared (IR) electronically controllable flare consisting of three distinct components: (1) a controllable pyrotechnical ejection block comprising three independently addressable small-scale propellers, all integrated into a one-piece molded and interconnected device, (2) a terminal function block comprising a structured IR pyrotechnical loaf coupled with a microinitiation stage integrating low-energy addressable pyroMEMS, and (3) a connected, autonomous, STANAG 4187 compliant, electronic sensor arming and firing block.

## 1. Introduction

In 1995, the pyroMEMS concept, which involves the integration of energetic material on an electronic chip, was introduced for medical applications using mechanical power derived from the combustion of a propellant [1] to inject drugs through the skin [2]. This original concept has led to major innovations and has inspired research that has defined the technological area called micropyrotechnics [3]. The subsequent fabrication of small pyrotechnic systems includes a wide range of applications: micropropulsion [4,5,6,7,8,9,10], microfluidics [11,12], electrical protection [13,14], in situ welding [13,14,15], safe arm and fire devices [16,17,18], and multipoint initiations [19,20]. The innovation of the pyroMEMS concept has been explored in several fields at many universities and research institutes: Berkeley University [21,22], Tohoku University [23], Georgia Tech [24], Sandia National Laboratory [25] and École polytechnique fédérale de Lausanne [11,26,27,28].

In the 2000s, pyroMEMS fabrication challenges revealed that is was necessary to replace conventional CHNO energetic materials with new, safer energetic materials compatible with MEMS. These new materials should feature extremely high amounts of stored chemical energy that can be released quickly and safely. Nanothermites containing nanoscale metallic fuel in contact with a strong oxidizer emerged as promising candidates because their burn rate can be tuned from mm/s to m/s, and even km/s in some particular cases [29,30,31]. To obtain a high interfacial contact area between the fuel and the oxidizer, ultrasonication [32], electrospraying/electrospinning [33], mechanical milling [34,35], self-assembly (static electricity-based [36], ligand-based [30,37,38], sol-gel [39] and DNA-based assembly [40,41,42] and, recently, 3D printing [43,44,45,46] approaches have been explored with varying levels of success. An alternative technique for creating high-density, high-interface surface area composites is utilizing nanolaminates, wherein nanosized layers of the oxidizer and the metal are deposited on top of each other using vacuum vapor deposition techniques [47,48]. These nanolaminate materials have a highly controllable architecture and are compatible with MEMS manufacturing processes [49,50,51,52,53,54,55]. Therefore, we anticipate that the progress made over the two last decades in both micro- and nanotechnologies and nanothermite materials will enable the integration of intelligence into pyrotechnical systems at the centimeter scale and smaller. In particular, to design new, miniature smart systems, we expect that pyrotechnic engineers will be able to rely on addressable pyroMEMS.

**Figure 1:**
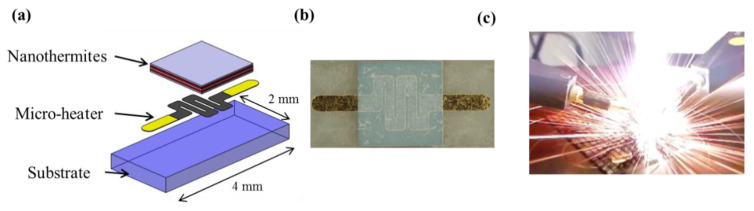
(**a**) A 3D schematic representation of a pyroMEMS used as an electric micro initiator, (**b**) a photo of the pyroMEMS, and (**c**) a photo taken during the nanothermite reaction.

In 2020, a pyroMEMS [56] consists of microscale electric initiators (Figure 1): a thin thermite layer is deposited on a thin-film resistive layer. When a current is applied to the resistance, the nanothermite is ignited by the Joule effect. The nanothermite then ignites a booster charge, the propellant, or the main energetic, which is usually called a secondary energetic. Because the pyroMEMS is manufactured using MEMS-based manufacturing techniques that are compatible with electronics, this component is low-cost while also maintaining high levels of performance and reliability due to its simple function. An additional advantage of replacing conventional “hot-wire” igniters or resistive bridge wires with pyroMEMS is that it eliminates the need for dangerous primary energetic formulations, such as lead styphnate, lead azide, and zirconium potassium perchlorate. Finally, pyroMEMS can be easily interconnected with electronics chips, which enables controllability over the ignition process.

In this paper, our goal is to illustrate how pyroMEMS and nanothermite materials can enable the integration of intelligence into centimeter-scale pyrotechnical systems by presenting the development of a smart and miniaturized infrared (IR) flare (1′ × 1″ × 8″). We chose IR flares application as they are essential safety elements and could be widely deployed by planes to counter an infrared homing missiles. Commonly, flares are composed of a magnesium based pyrotechnic composition with burning temperature hotter than the plane engine exhaust. Among IR flares, smart IR flares integrate decision and action capacities and should be able to adapt their pyrotechnical response to efficiently protect different vehicles and perceive various threats. To achieve these goals, we integrate addressable pyroMEMS as microinitiators to regulate the IR effect. The paper is organized as follows: we first present the flare design, technological choices, and prototyping for each block before describing the assembly of a representative IR flare.

## 2. Smart Flare Design

**Figure 2:**
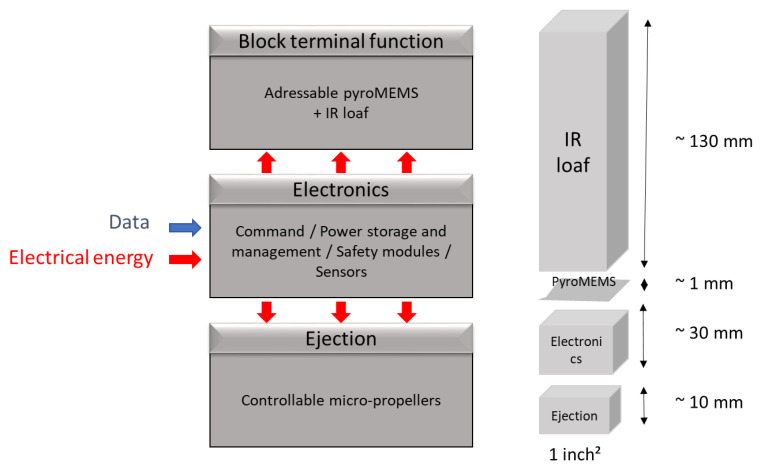
Smart IR flare structure: (**left**) block diagram and (**right**) the corresponding dimensions.

The IR flare design consists of three main functional blocks that are mechanically and electronically interconnected (Figure 2): (1) a controllable pyrotechnical ejection block comprising three independently addressable small-scale ejectors, also called propellers, (2) a terminal function block comprising a structured IR pyrotechnical loaf coupled with a low-energy and addressable pyroMEMS ignition stage, and (3) a connected, autonomous STANAG 4187 compliant electronic sensor arming and firing block (this component is labeled as “electronics” in Figure 2).

First, the *intelligence* checks the validity of each electronic and pyrotechnical component. If everything passes the validity check, the electrical energy is used to power all the system parts. Then, the flare ejection from the shuttle or plane is triggered, where the ejection speed is controlled by the number of propellers ignited. After a period of time that is preset by the user, usually a few seconds, the safeties are unlocked and the IR loaf is ignited using one, two or more pyroMEMS, depending on the desired IR signature.

## 3. Block Fabrication and Testing

### 3.1. Ejection Blocks Based on Addressable Impellors

**Figure 3:**
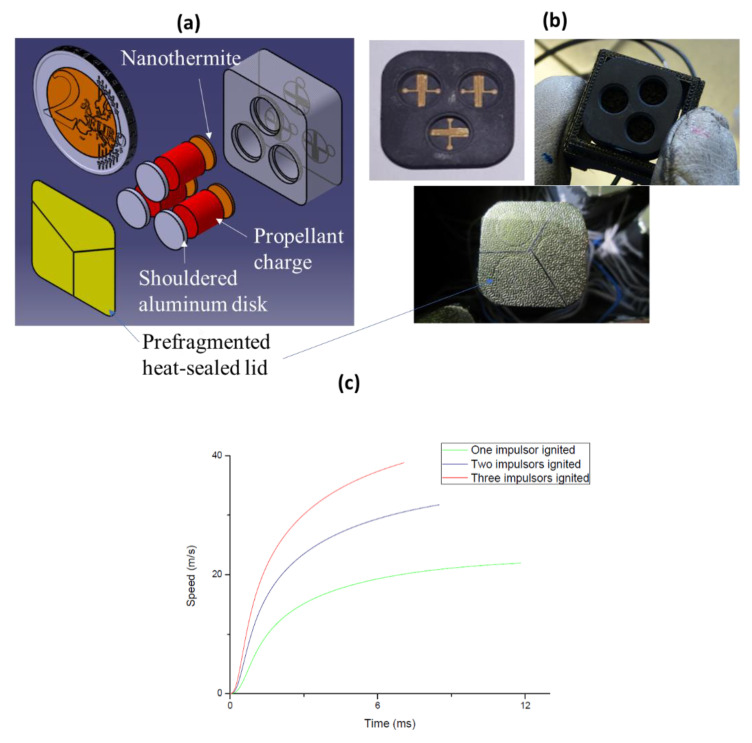
(**a**) Schematic of the triple plastronic impulsor concept made in MID technology, (**b**) a serie of photos of one metalized plastic prototype made of 3 impulsors (one is filled with propellant and sealed with the heat-sealed lid), and (**c**) the ejection speed as a function of the configuration: one, two or three ignited impulsors.

To increase the applicability of smart IR flares, our first objective is to control their tube ejection speed. To this end, we propose and design a multi-impellor concept that integrates independent propellant charges. The number of charges ignited thus determines the ejection speed. We use molded interconnect device technology [57], also called plastronic technology, to metalize the plastic 3D parts (Figure 3). For demonstration purposes, we restrict the scope of our study to three identical charges (Figure 3b). The thin-film resistor is patterned onto the bottom of the cavity, which is then filled with nanothermites.

We implement a STANAG 4367-based lumped parameter internal ballistics model to choose the ejection part size. We conduct a response surface methodology study [2] to extract the conception parameters corresponding to minimal ejection charges for given ejection speeds. These parameters include combustion and expansion chamber volumes, cartridge stopper and ejector lid unsealing pressures. We clearly see on Figure 3c, that the ejection speed, i.e., the speed at which the pyrotechnical loaf is ejected from the cartridge ranges from 20 to 40 m/s by choosing to fire one, two or three impulsors.

We manufacture the prototypes and start by molding the 3 cm^3^ plastic parts, in which we bore cylindrical chambers (Figure 3b). We use a laser beam to activate the selected surfaces then dip the parts in metal-oxide baths, creating metalized hot wires on the bottom of each chamber and communication tracks on the outer sides of each part. In each chamber, we deposit Al/CuO nanothermites, acting as an ignition charge, and bore-potassium nitrate grains, acting as propellants. Finally, we close each chamber with a shouldered aluminum disk then hot-seal a prefragmented aluminized polymer lid on top of the plastic piece to ensure air and hot-gas tightness. Compared to existing copper and glass ejectors with soldered nichrome hot wires, using plastronic impellors reduces the interfacial and assembly complexity. In particular, plastronics enable several combustion chambers to interface with electronics in small volumes, therefore allowing for controlled ejection in 1 inch-squared flares.

We validate the triple plastronic impellor prototypes in open air, in closed bombs and in representative firings. The prototypes are fully functional but exhibit higher initiation delays than existing Ni-Cr hot-wire igniters (11.2 ms compared to 5 ms for 5 A/1 Ω). These initiation delays could be easily improved by optimizing the resistive film. The ejection speed can also be tuned from 20 to 40 m/s when one, two or three impellors are ignited (Figure 3c).

### 3.2. The IR Loaf Block

**Figure 4:**
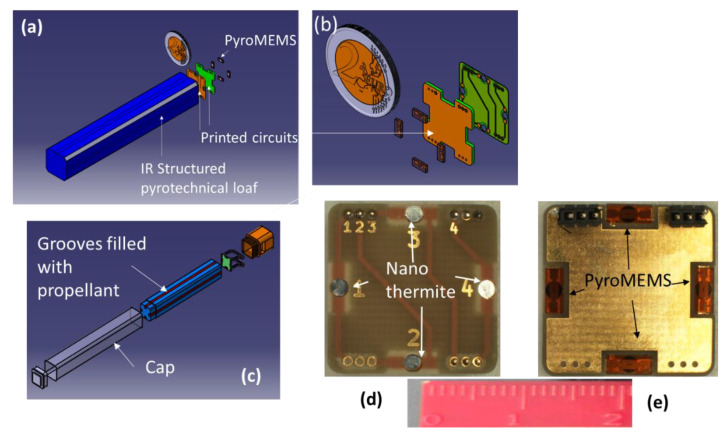
(**a**) Schematic of the IR loaf comprising 4 grooves (blue) filled with IR pyrotechnical charges that can be ignited using the 5 × 2.2 mm pyroMEMS mounted on the PCBs (green). (**b**) Enlarged view of the microinitiation stage comprising 4 addressable pyroMEMS mounted on the PCBs. (**c**) Enlarged view of the pyrotechnical loaf components. (**d**) Photo of the front side of the PCB, which is in contact with the IR loaf, where the nanothermites can be distinguished. (**e**) Photo of the back side of the PCB, where the pyroMEMS can be distinguished.

**Figure 5:**
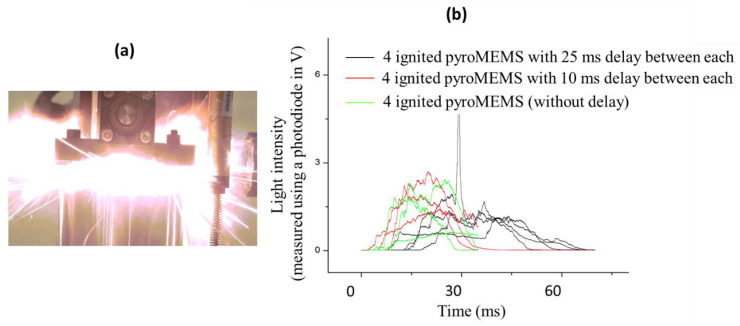
(**a**) Photo of the flame produced by the propellant used for demonstration, which is made of magnesium/Teflon/Viton and was ignited by four pyroMEMS. (**b**) Light emitted by the IR flare, with curves representing the different numbers of grooves ignited by the pyroMEMS. Black: 4 pyroMEMS sequentially ignited with a delay of 25 ms. Red: 4 pyroMEMS sequentially ignited with a delay of 10 ms. Green: 4 pyroMEMS ignited together.

The second objective is to control the IR signal emitted by the combustion of a pyrotechnic loaf so that a single flare can emulate the IR signature for various vehicles as seen from various angles. To control this signal, we design a micro multipoint-initiation stage coupled to a structured pyrotechnical loaf. More precisely, we couple an addressable 5 × 2.2 mm pyroMEMS [53,58] (Figure 4) with initiation-composition-filled grooves on the sides of a metal-polymer pyrotechnical loaf, whose surfaces are coated with an inert epoxy-based resin (Figure 4d,e). We inert the surfaces of the loaf, using the “cap” in Figure 4c, and ignite the loaf either sequentially or partially using millimeter-scale ignition points. In doing this, we ensure electronic control of the independent combustion front generation and, thus, control the corresponding IR effect. Once again, for demonstration purposes, we integrate four pyroMEMS coupled to four corresponding grooves, one on each side of a parallelepiped loaf (Figure 4a,c). We experimentally validated the ignition of a common initiation source made of magnesium/Teflon/Viton. Each pyroMEMS ignites reliably (100% success) within 0.7 ms under a 1 A secondary energetic composition source. Less sensitive source compositions, such as glycidyle azide polymer-based propellant ignite in 10 to 300 ms under 1 A, depending on their sensitivities. The measured ignition delays using nanothermites are equivalent to those observed on existing flares, where hot, pressured gases ignite the initiation sources. A photo taken during one ignition test is shown in Figure 5a.

We also confirmed that the IR signal light intensity varies between the factors of one and three, the signal duration varies between the factors of one to two and the maximum light intensity varies between the factors of one and two, depending on the initiation sequence (Figure 5b). The light produced by the pyrotechnical loaf lasts 57.5 ms when pyroMEMS are ignited sequentially with 25 ms delay between each. The light produced by the pyrotechnical loaf lasts 30 ms in the two other cases, i.e., when the four pyroMEMS are ignited in one single sequence or with a delay of 10 ms. We conclude that a sequencing of 10 ms is not sufficient to see an effect of the IR emission.

### 3.3. Electronics

**Figure 6:**
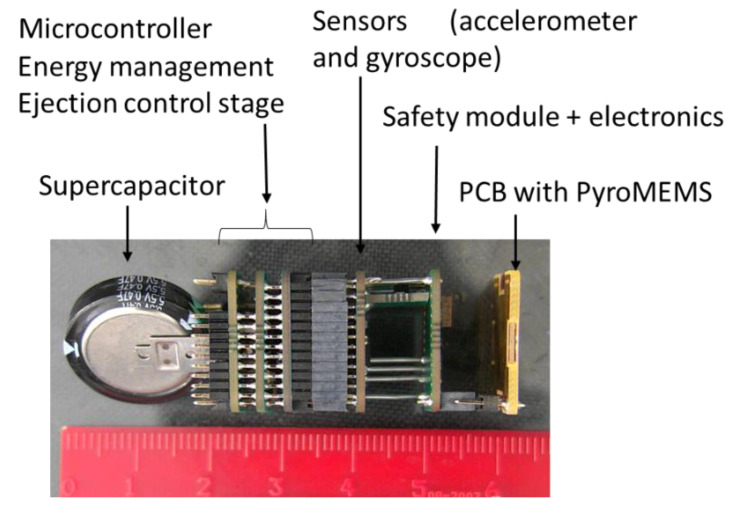
Photo of the electronics. In red, below the device, is a ruler to indicate the dimensions.

To control the micro initiation sequence and ensure safety, the smart flare is integrated with its own electronic module. The main smart flare functions include self-testing, controlling, arming and firing, detecting ejection through the embedded accelerometer and gyroscope sensors, communicating with the launcher/plane, and managing the electrical energy. The electronic circuitry comprises five interconnected printed board circuits (PCBs). Regarding safety features, we arbitrarily choose to comply with STANAG 4187 (See Supplementary file), which is designed for warheads, to prove that, given our dimensional constraints, we could comply with any given security requirements. For energy storage, our prototype integrates less than 25 cm^3^ of a 0.47F SG supercapacitor from Panasonic. A PIC microcontroller enables control/command and communication on a CAN bus and microcurrent sources from LinearTech to reproducibly ignite the MEMS initiators. To detect nominal ejection and to trigger arming, the circuitry also integrates a 6050 IMU from InvenSense and a VCNL4040 IR emitter/receptor from Vishay (Figure 6).

### 3.4. Device Integration and Concept Validation

**Figure 7:**
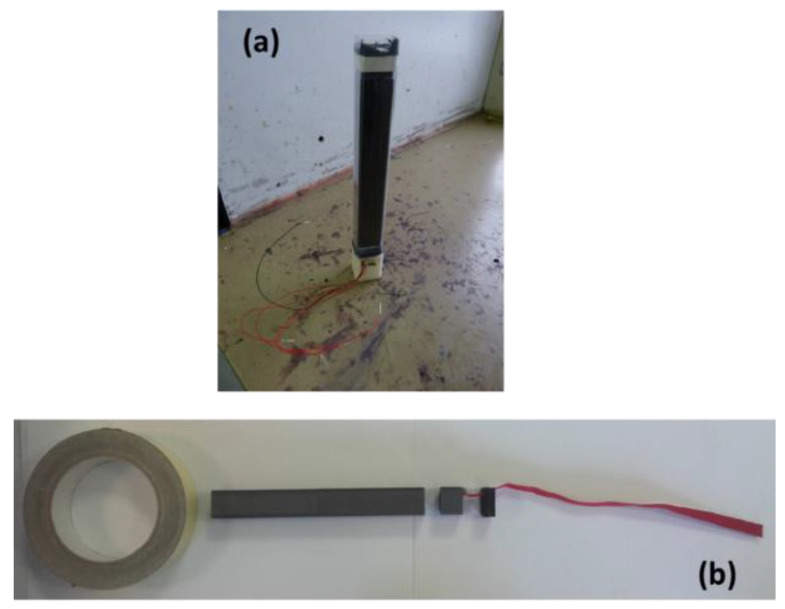
Smart IR flare prototypes (**a**) using conventional electrical wires for testing. (**b**) using electrical wrapped connectors.

To avoid overshadowing the advantages of the aforementioned technological blocks, we paid careful attention to their interfaces and integration. First, to ensure backward launcher compatibility, we route the energy and data through the bottom of the cartridge, where the ejection block lies. This implies that the conductive tracks have to cross the ejection block before reaching the electronics (see Figure 2). To foster miniaturization, integration and reliability, we use plastronics to metalize four tracks upon the ejection block, two for CAN communication and two for electrical power transfer. Second, for the pyroMEMS, the electric pads and the thermite nanolaminate are placed on the same side of the chip. Until now, the pyroMEMS interface required wire bonding or conductive adhesive. We soldered the chips onto PCBs with holes before encapsulation to enable the system to endure harsher climatic and mechanical conditions and to improve electronic integration. Last, because of their reliability and relatively small size, we used standard pin headers for the interconnections between the PCBs. Photos of the smart IR flare prototype is provided in Figure 7.

As the goal of this research was to demonstrate that the use of pyroMEMS and associated nanothermites materials instead of conventional pyrotechnical charges, enables both the miniaturization and integration of intelligence into flares, we assembled five demonstrators and tested them following different sequences. We could demonstrated that (1) the flare ejection speed can be tuned from 20–40 m/s by employing plastronic technology (MID) and nanothermite instead of conventional technology. (2) The emitted light intensity and duration can be also tuned by simply sequencing the pyroMEMS ignition. We also succeeded in the miniaturization purpose as all functions are included into 1 inch^2^ by 8 inches. Next step, taken in charge by the industrial partner will be the characterization of IR signature as well as addressing the reliability issues.

## 4. Conclusions

Until now, the size and energy consumption of existing hot-wire igniters have limited their integration into miniature smart energetic systems. Using pyroMEMS no larger than 8 mm^3^ and consuming less than 3 mJ to ignite, we integrate a large number of initiators in the same volume, which allows us to finely tune the final pyrotechnical effect. In this paper, we demonstrated the feasibility of a controllable, autonomous, safe and smart 1″ × 1″ × 8″ IR flare. We have therefore developed innovative technological bricks that enable control over both the flare ejection velocity and the IR effect using an innovative multipoint-initiation concept. Namely, we developed a plastronic-based triple impellor and a microinitiation stage powered by a supercapacitor to ignite an IR loaf after ejection. In doing this, it was essential to use novel initiators and integration techniques to meet ambitious miniaturization and functionalization requirements.

## Supplementary Materials

The following are available online at https://www.mdpi.com/2072-666X/12/2/118/s1.
