# Supplementary material for: PyroMEMS as Future Technological Building Blocks for Advanced Microenergetic Systems
Source: Micromachines (Basel). 2021 Jan 23;12(2):118. doi: 10.3390/mi12020118 (PMC7911588; doi:10.3390/mi12020118)
Supplement: Supplementary file 1 [file micromachines-12-00118-s001.pdf]

NATO | OTAN
NATO STANDARDIZATION AGENCY
AGENCE OTAN DE NORMALISATION
NSA

DECLASSIFIED - PUBLICLY DISCLOSED - PDN(2016)0019 - DÉCLASSIFIÉ - MIS EN LECTURE PUBLIQUE

09 March 2006 NSA/0265(2007)-JAS/4187

See CNAD AC/326 STANAG distribution

STANAG 4187 JAS (EDITION 4) – FUZING SYSTEMS: SAFETY DESIGN REQUIREMENTS

Reference: PFP(AC/326)D(2003)002 dated 15 July 2003

1. The enclosed NATO Standardization Agreement, which has been ratified by nations as reflected in the **NATO Standardization Document Database (NSDD)**, is promulgated herewith.

2. The reference listed above is to be destroyed in accordance with local document destruction procedures.

ACTION BY NATIONAL STAFFS

3. National staffs are requested to examine **their ratification status of the STANAG** and, if they have not already done so, advise the Defence Investment Division through their national delegation as appropriate of their intention regarding its ratification and implementation.

J. MAU
Major General, POL(A)
Director, NSA

Enclosure:
STANAG 4187 (Edition 4)

North Atlantic Treaty Organisation – Organisation du Traité de l'Atlantique Nord
B-1110 Brussels Belgium
E-mail: mc.mortier@hq.nato.int – Tel 32.2.707.3942 – Fax 32.2.707.4103

DECLASSIFIED - PUBLICLY DISCLOSED - PDN(2016)0019 - DÉCLASSIFIÉ - MIS EN LECTURE PUBLIQUE

NORTH ATLANTIC TREATY ORGANIZATION
(NATO)

NATO STANDARDIZATION AGENCY
(NSA)

STANDARDIZATION AGREEMENT
(STANAG)

SUBJECT: FUZING SYSTEMS - SAFETY DESIGN REQUIREMENTS

Promulgated on 09 March 2007

J. MAJ
Major General, POL(A)
Director, NSA

RECORD OF AMENDMENTS

No.	Reference/Date of amendment	Date entered	Signature

EXPLANATORY NOTES

AGREEMENT

1. This NATO Standardization Agreement (STANAG) is promulgated by the Director NATO Standardization Agency under the authority vested in him by the NATO Standardization Organisation Charter.

2. No departure may be made from the agreement without informing the tasking authority in the form of a reservation. Nations may propose changes at any time to the tasking authority where they will be processed in the same manner as the original agreement.

3. Ratifying nations have agreed that national orders, manuals and instructions implementing this STANAG will include a reference to the STANAG number for purposes of identification.

RATIFICATION, IMPLEMENTATION AND RESERVATIONS

4. Ratification, implementation and reservation details are available on request or through the NSA websites (internet http://nsa.nato.int; NATO Secure WAN http://nsa.hq.nato.int).

DECLASSIFIED - PUBLICLY DISCLOSED - PDN(2016)0019 - DÉCLASSIFIÉ - MIS EN LECTURE PUBLIQUE

DECLASSIFIED - PUBLICLY DISCLOSED - PDN(2016)0019 - DÉCLASSIFIÉ - MIS EN LECTURE PUBLIQUE

NATO STANDARDIZATION AGREEMENT (STANAG)

FUZING SYSTEMS - SAFETY DESIGN REQUIREMENTS

Annexes:

A National Safety Approving Authorities.
B Definitions.
C Safety Design Requirements for Naval and Land Service Mine Fuzing Systems.
D Pyrotechnic Initiated Explosive Projectiles.

Related documents
The following documents of the date of issue of this document form the basis for or have relationship to this document.

Related Referenced Documents:

STANAG 1307	Maximum NATO Naval Operational Electromagnetic Environment Produced by Radio and Radar.
STANAG 4145	Nuclear Survivability Criteria for Armed Forces Materiel and Installations.
STANAG 4147	Chemical Compatibility of Ammunition Components Explosives and Propellants (Non Nuclear Applications).
STANAG 4157	Fuzing Systems: Test Requirements for the Assessment of Safety and Suitability for Service.
STANAG 4170	Principles and Methodology for the Qualification of Explosive Materials for Military Use.
STANAG 4234	Electromagnetic Radiation (Radio Frequency) 200 kHz to 40 GHz Environment Affecting the Design of Materiel for Use by NATO Forces.
STANAG 4235	Electrostatic Environmental Conditions Affecting the Design of Materiel for Use by NATO Forces.
STANAG 4236	Lightning Environmental Conditions Affecting the Design of Materiel for use by NATO Forces.
STANAG 4239	Electrostatic Discharge, Munitions Test Procedures.
STANAG 4297	Guidance on the Assessment of the Safety and Suitability for Service of Munitions for NATO Armed Forces. – AOP 15
STANAG 4324	Electromagnetic Radiation, (Radio Frequency) Test Information to Determine the Safety and Suitability for Service of Electro- Explosive Devices and Associated Electronic Systems in Munitions and Weapon Systems.

STANAG 4327	Lightning, Munition Test Procedures.
STANAG 4363	Fuzing Systems - Development Testing for the Assessment of Lead and Booster Explosive Components.
STANAG 4370	Environmental Testing
STANAG 4416	Nuclear Electromagnetic Pulse, Test Procedures.
STANAG 4423	Cannon Ammunition Safety and Suitability for Service Assessment
STANAG 4518	Safe Disposal of Munitions, Design Principles and Requirements, and Safety Assessment.
STANAG 4560	Electro-Explosive Devices, Assessment and Test Methods for Characterisation
AOP-15	Guidance on the Assessment of the Safety and Suitability for Service of Munitions for NATO Armed Forces.
AOP-16	Fuzing Systems - Design Guides.
AOP 42	Integrated Design Analysis for Fuzing and Safety Critical Systems
AECP-1	Mechanical Environmental Conditions to which Materiel Intended for Use by NATO Forces could be Exposed.
AQAP-110	NATO Quality Assurance Requirements for Design, Development and Production.
AECTP-500	Electrical Environmental Test.

Related Information Documents:

STANAG 4369	Design Requirements for Inductive Setting of Large Calibre Projectile Fuzes.
STANAG 4423	Cannon Ammunition Safety and Suitability for Service Assessment
AOP-7	Manual of Tests for the Qualification of Explosive Materials for Military Use.
AOP-8	NATO Fuze Characteristics Data.
AOP 20	Manual of Tests for the Safety Qualification of Fuzing Systems
AOP-21	Fuzing Systems: Manual of Development Characterisation and Safety Test Methods and Procedures for Lead and Booster Explosive Components.

DECLASSIFIED - PUBLICLY DISCLOSED - PDN(2016)0019 - DÉCLASSIFIÉ - MIS EN LECTURE PUBLIQUE

DECLASSIFIED - PUBLICLY DISCLOSED - PDN(2016)0019 - DÉCLASSIFIÉ - MIS EN LECTURE PUBLIQUE

AOP 43	Electro-Explosive Devices - Assessment and Test Methods for Characterisation Guidelines for STANAG 4560
AECTP-100	Environmental Testing - Guidelines on Management Planning.
AECTP-200	Environmental Testing - Definitions of Environments.
AECPT-300	Climatic Environmental Tests.
AECPT-400	Mechanical Environmental Test.

AIM

1. The aim of this agreement is to standardize safety design requirements for fuzing systems for operational and training munitions for use by NATO Forces.

AGREEMENT

2. Participating nations agree to design munition fuzing systems in accordance with the requirements of this STANAG. The agreement applies as follows:

a. Applicability. The agreement is applicable to the development of new fuzing systems, commenced after promulgation of this STANAG, for all munitions except those listed in Paragraph 2.b. The design requirements of this STANAG shall be applied to the design of Mission Termination Systems (MTS) where such systems work by initiating the munition or submunition warhead(s). When the MTS uses a break-up charge, the design shall be referred to the NSAA for approval that the system provides sufficient safety. This agreement shall be applied by the National Safety Approving Authorities (NSAA) listed in Annex A.

b. Exclusions. The following munitions are **EXCLUDED** from this agreement:

(1) Nuclear weapon systems and their associated training aids.

(2) Flares and Signals dispersed only by hand.

(3) Pyrotechnic Countermeasure Devices.

(4) Those munitions that are recognised by the NSAA as being designed to detonate, deflagrate or disperse without constraints on safety at intended functioning.

(5) Munitions which the NSAA agrees do not present sufficient hazard as to require a fuzing safety system.

(6) Hand Emplaced Munitions.

DEFINITIONS

3. Definitions of terms which are specific to this STANAG are given at Annex B with further definitions related to the states fuzing systems may adopt given at Annex B, Appendix 1.

DECLASSIFIED - PUBLICLY DISCLOSED - PDN(2016)0019 - DÉCLASSIFIÉ - MIS EN LECTURE PUBLIQUE

GENERAL

4. **DESCRIPTION**

A fuzing system includes all devices that fulfill the following:

a. Ensures the safety of the initiation system of the munition payload throughout the logistic phases and operational usage as well as testing and inspection.

b. Recognizes or determines the circumstances under which the munition payload is intended to function.

c. Enables and initiates the munition's payload.

d. Where applicable, recognises or determines the circumstances under which the munition payload is intended to be de-armed, sterilised or to self-destruct.

5. **GENERAL CONSIDERATIONS**

a. Specific Classes. Safety design requirements for the fuzing systems of mines and Pyrotechnically Initiated Explosive (PIE) projectiles are stated in the following Annexes:

Annex C: Safety Design Requirements for Mine Fuzing Systems.

Annex D: Pyrotechnic Initiated Explosive Projectiles.

b. Design Approval. During the concept development phase, the developing agency shall obtain approval from the NSAA for both the design concept and the methodology for assuring compliance with safety requirements. At the completion of engineering development, the developing authority shall present a Design Safety Assessment to the NSAA for review to obtain approval of the design (See Paragraph 14.c).

c. Safety Design Requirements.

(1) A life cycle environmental profile shall be defined for the fuzing system which will establish the environmental conditions and limits the fuzing system will encounter throughout its life cycle.

(2) The fuzing system shall be designed to maintain the required degree of safety in credible accident situations and under all specified natural and induced environmental conditions in its life cycle. (See STANAGs 1307, 4157, 4234, 4235, 4236, 4297 and AECP-1).

d. Application. The features, requirements, procedures and controls listed in this Agreement shall be used in the design of fuzing systems.

e. Guidance. Guidance on the interpretation of the requirements stated in this STANAG is given in AOP-16.

DECLASSIFIED - PUBLICLY DISCLOSED - PDN(2016)0019 - DÉCLASSIFIÉ - MIS EN LECTURE PUBLIQUE

DETAILS OF THE AGREEMENT

6. FUNDAMENTAL SAFETY DESIGN REQUIREMENTS

The following safety design requirements shall apply to all fuzing systems:

a. Inclusion of Safety Features.

(1) Fuzing systems shall include at least two safety features. The control and operation of these safety features are to be functionally isolated from other processes within the munition system and each of which shall prevent unintentional arming of the fuzing system. At least two of the safety features shall be independent and designed to minimise the potential for common cause failure.

(2) Where it is not technically possible to functionally isolate the safety features, those non-isolated components, including software, used to enable the safety features shall be considered part of the fuzing system and must meet the requirements of this STANAG. The reason for not complying with paragraph 6.a.1. and mitigation must be provided to the NSAA that the safety requirements have still been maintained.

(3) At least one of the independent safety features shall prevent arming after launch or deployment until the specified safe separation distance or equivalent delay has been achieved.

b. Operation of Safety Features Using Environmental Stimuli.

(1) The stimuli which enable the independent safety features to operate shall be derived from different environments or different combinations of environments or both; where combinations are used each combination shall be different.

(2) The environments selected and sensed by the fuzing system to remove safety features during arming shall avoid any environment or levels of environmental stimulus that may be experienced by the fuze prior to the commencement of the launch cycle.

(3) Operation of at least one of the independent safety features shall depend on sensing an environment after first motion in the launch cycle or on sensing a post launch environment.

(4) Any action taken to launch a munition may be considered an environmental stimulus if it irreversibly commits the munition to complete the launch cycle.

(5) Munitions that are designed to be jettisoned shall not have safety compromised by the action of such release.

c. Prevention of Unintentional Arming.

(1) Safety design shall ensure that:

(a) Fuzing systems are not capable of being armed manually.

(b) Fuzing systems do not rely solely upon defined operating drills or procedures to provide safety.

(2) Fuzing systems shall be designed so that no single credible circumstance can result in arming before launch or deployment.

(3) Fuzing systems shall not be capable of arming except as a consequence of a sequence of actions resulting from the sensing of environments that occur during or after launch or deployment.

(4) Fuzing systems shall use environmentally derived energy generated after initiation of the launch or deployment cycle in preference to pre-launch stored energy to enable or arm the system. If this cannot be practically achieved and stored energy is used, then the system safety hazard analyses shall demonstrate that no failure mode for that source of energy will compromise the specified failure probabilities for that system (See Paragraph 14).

(5) Fuzing system features which control arming, including safety logic devices and safety logic shall be dedicated to the control of arming.

d. Application of Requirements to Multiple Safety and Arming Devices. The requirements at Paragraphs 6.a to 6.c apply to all munitions, including sub munitions, having a single safety and arming device. For munitions with multiple safety and arming devices compliance shall be as follows:

(1) Independent Safety and Arming Devices. When a fuzing system incorporates multiple safety and arming devices for which the functions of arming and initiation are independent, the requirements at Paragraphs 6.a to 6.c shall apply to each safety and arming device.

(2) Interrelated Safety and Arming Devices. When a fuzing system incorporates multiple safety and arming devices, which share common functions of arming, initiation or both, the requirements at Paragraphs 6.a to 6.c shall apply overall to the interrelated safety and arming devices.

(3) The system shall be designed so that no sub-munition fuze can arm solely as a result of dispersing from the carrier munition during a credible accident before launch of the carrier munition.

e. Fuze Setting. If fuze setting is safety critical (e.g. arming time, function time, or proximity broadcast turn on time) uncontrolled alteration of the set value shall be prevented.

f. Fail Safe Design. Fuzing systems shall incorporate fail-safe design features based on their applicability to system requirements.

g. Self-Destruction, Sterilisation, De-arming. Self-Destruct may take one of two forms: self-functioning or self-disruption. If required in the system requirement document, a self-destruct, sterilisation and/or de-arming feature may be included. None of these shall increase the probability of hazards to the operator, over those that exist from the same munition fitted with a fuzing system without such a feature. Self-destruction shall not be initiated prior to launch and achieving safe separation distance or equivalent arming delay.

h. Disposal. Fuzing systems shall meet the requirements of STANAG 4518.

i. Single Device. The elements of the fuzing system that prevent arming until valid launch environments have been sensed and the arming delay has been achieved should be located in a single safety and arming device.

DECLASSIFIED - PUBLICLY DISCLOSED - PDN(2016)0019 - DÉCLASSIFIÉ - MIS EN LECTURE PUBLIQUE

DECLASSIFIED - PUBLICLY DISCLOSED - PDN(2016)0019 - DÉCLASSIFIÉ - MIS EN LECTURE PUBLIQUE

7. EXPLOSIVES IN FUZING SYSTEMS

Explosive compositions and materials shall be selected as follows:

a. Assessment and Qualification of Explosives. Explosives and explosive compositions shall be assessed and qualified in accordance with the requirements of STANAG 4170 in their design role (primary, lead, booster, expulsion charges, etc.).

b. Safety in Storage and Use. Explosive compositions shall be chosen, so that the system is safe and remains so under the specified conditions of storage and use.

c. Sensitiveness. The sensitiveness of the explosives shall not increase significantly during the entire service life of the fuze beyond the level at which they were approved for service use.

d. Qualification and Sensitiveness of Expulsion Charges and Lead and Booster Explosives. Only those explosives Qualified in accordance with the requirements of STANAG 4170 as acceptable expulsion charges and lead or booster explosives are permitted to be in a position leading to the initiation of a high explosive main charge without interruption. The explosive material used in fuzing systems shall not be altered by any means likely to increase its sensitiveness beyond that at which the material was qualified.

e. Assessment of Explosive Components. Lead and booster explosive components in fuzing systems shall be assessed in accordance with the requirements of and pass the tests specified in STANAG 4363.

8. CONTROL OF EXPLOSIVE TRAINS IN FUZING SYSTEMS

a. Use of Interrupted Explosive Trains. When the explosive train contains primary explosives or explosives other than those allowed by Paragraph 7.d, the train is to be interrupted and the following requirements shall apply:

(1) At least one interrupter (barrier, shutter, slider, rotor) shall isolate the primary explosive and/or explosives that do not meet the requirements of Paragraph 7.d, from subsequent elements of the explosive train. The interrupter(s) shall be directly locked mechanically in the safe position by at least two independent safety features of the fuzing system until the start of the arming sequence.

(2) The interrupter shall prevent propagation of an explosive event to any acceptor explosive element, contained within the explosive train after the interrupter, until the required safe separation or equivalent delay has lapsed. The explosive train interruption shall be evaluated by the Primary Explosive Component Safety Test as given in STANAG 4157.

(3) Designs in which the primary explosive is positioned such that safety is completely dependent upon the presence of an interrupter shall include positive means to prevent the fuzing system from being assembled if the interrupter is excluded or if the interrupter is in the unsafe position.

b. Use of Non-Interrupted Explosive Trains. Explosive train interruption is not required when only those explosive materials allowed by Paragraph 7.d are used in the train. In these circumstances one of the following methods of controlling arming shall be used:

(1) For fuze systems using techniques for accumulating all functioning energy from the post-launch environment, the system shall prevent arming until verification, by the system, of a proper launch and attainment of the required arming delay. Accumulation of any functioning energy shall not occur until as late in the arming cycle as operational requirements permit.

(2) For fuze systems using techniques that do not accumulate all functioning energy from the post-launch environment, the following shall apply:

(a) At least two safety features (that meet the requirements of Paragraph 6) shall enable at least 3 energy breaks.

(b) At least one energy break shall be capable of preventing arming in a static mode if any or all of the energy breaks are left out or malfunction. This requires at least one energy break to function in a dynamic mode.

(c) At least one energy break shall function in a static mode.

(d) Independent control of energy breaks shall be exercised to the maximum extent possible; a minimum of two separate logic devices shall be employed.

9. **ELECTRICAL INITIATORS AND ELECTROEXPLOSIVE DEVICES (EEDs).**

a. Shall be characterised in accordance with STANAG 4560 and that information shall be made available to the NSAA.

b. Shall be qualified to specific test procedures and pass/fail criteria established or approved by the NSAA.

c. Electrical initiators used in non-interrupted explosive trains shall:

(1) Not be capable of being detonated by any electrical potential of less than 500 V applied directly to the initiator.

(2) Not be capable of being initiated by an electrical potential of less than 500 V when applied to any accessible part of the fuzing system during and after installation into the munition or any munition subsystem.

10. **ADDITIONAL SAFETY DESIGN REQUIREMENTS FOR FUZING SYSTEMS CONTAINING ELECTROMECHANICS AND ELECTRONICS**

The following safety design requirements shall apply to electromechanical and electronic fuzing systems in addition to those given elsewhere in this STANAG:

a. EED No-Fire Threshold Safety Margins. In any safety and arming system in which safety is dependent on preventing the unintentional functioning of an EED, a minimum safety margin between the no-fire threshold (NFT) stimulus and the stimulus that could be induced by electrical or electromagnetic interference shall be demonstrated to and accepted by the NSAA.

b. Arming and Initiation. Designs shall ensure that:

(1) Independent safety feature controls (e.g., logic) are physically separated and implemented using different component types to minimise the potential for common cause failures.

DECLASSIFIED - PUBLICLY DISCLOSED - PDN(2016)0019 - DÉCLASSIFIÉ - MIS EN LECTURE PUBLIQUE

(2) Where Built-In Test (BIT) or other in-service tests on the integrity of the fuze are required, the safety of the fuze shall not be degraded.

c. Electrical Firing Energy Dissipation. For electrically initiated fuzing systems, the design shall include a provision to deplete the firing energy after the operating lifetime of the fuzing system has expired. The time required to dissipate the firing energy shall be reduced to the minimum allowed by the operating requirements for the fuzing system. The means of dissipation shall be designed so that it does not degrade the overall safety of the Safety and Arming device before the system is armed.

d. Safety Critical Computing Systems. The safety design requirements and guidelines as stipulated by the NSAA shall be complied with. Requirements for safety logic features are as follows:

(1) Information Transfer. Information passed between an environmental sensor and an arming system shall be transferred by a defined logic route dedicated to that transfer only.

(2) Interpretation of Information. Information received by the arming system shall be capable of being verified as a valid command to begin a sequence of events resulting in the removal of a safety feature. False or corrupted data shall not cause the removal of a safety feature.

(3) Computing Systems. Non-embedded software shall not be used. If a computing system with embedded software is used to perform the logic function, then it shall be designed to facilitate a safety assessment to the satisfaction of the NSAA.

e. Hardware Excluding Computing Systems. If the logic function is performed by totally dedicated hardware to give unequivocal interpretation, the hardware systems shall use components in which all the logic states can be identified, verified and validated. The design selected shall be approved by the NSAA.

f. Electromagnetic, Lightning and Electrostatic Immunity.

(1) Electromagnetic Immunity. The fuzing system shall not exhibit unsafe operation during and after exposure to electromagnetic energy including lightning. Immunity from arming or firing due to electromagnetic forms of energy shall be demonstrated by analyses and appropriate tests as necessary, which duplicate or simulate credible life-cycle electromagnetic environments. (See STANAGs 4234/4324 (EMR), 4236/4327 (Lightning Effects), 4145/4416 (EMP) and 4370, AECTP 500 series (EMC)). Electro-magnetic interference should be tested in accordance with tests approved by the NSAA (STANAG 4157).

(2) Electrostatic Immunity. The fuzing system shall not exhibit unsafe operation during or after exposure to an electrostatic environment. Immunity from arming or firing due to electrostatic forms of energy shall be demonstrated by analyses and appropriate tests as necessary, which duplicate or simulate credible life-cycle electrostatic environments. (See STANAG 4235/4239/4157).

DECLASSIFIED - PUBLICLY DISCLOSED - PDN(2016)0019 - DÉCLASSIFIÉ - MIS EN LECTURE PUBLIQUE

DECLASSIFIED - PUBLICLY DISCLOSED - PDN(2016)0019 - DÉCLASSIFIÉ - MIS EN LECTURE PUBLIQUE

11. **COMPATIBILITY OF COMPONENTS**

All components used in the fuzing system shall be chosen to be compatible and stable so that under all specified natural and induced environmental conditions in its life cycle, none of the following can occur in an unarmed fuzing system:

a. Premature arming or functioning.

b. Dangerous ejection or exudation of material.

c. Deflagration or detonation of the lead or booster.

d. The formation of dangerous or incompatible compounds. Material which could contribute to the formation of more volatile or more sensitive compounds should not be used. If used, then the material shall be treated, located or contained to prevent the formation of a hazardous compound (see STANAG 4147).

e. Production of unacceptable levels of toxic or other hazardous materials.

f. A compromise of the safety, de-arming, sterilisation or self-destruct features, e.g., by electro-chemical reaction.

12. **SAFETY DESIGN REQUIREMENTS TO PROVIDE NON-ARMED ASSURANCE DURING ASSEMBLY AND INSTALLATION**

a. To provide non-armed assurance, fuzing system designs shall incorporate one or more of the following:

(1) A feature which prevents the assembly of the fuzing system in an armed condition.

(2) Positive, direct and unambiguous means of determining that the fuzing system is not armed during and after assembly and when installing the system into a munition. Where the fuzing system is accessible after assembly into the munition, the positive means of determination shall also be available. For fuzing systems with non-interrupted explosive trains, the method used shall positively prevent the accumulation of energy, of the type used for arming, in the system prior to installation in the munition. Any means employed in compliance of this paragraph shall not degrade safety.

(3) A feature which prevents installation of an armed fuzing system into a munition.

b. If arming and disarming of the assembled fuzing system in tests is a normal procedure in manufacturing, inspection, or at any time prior to its installation into a munition, the adoption of Paragraph 12a.(1) alone is not sufficient and either Paragraph 12a.(2) or Paragraph 12a.(3) shall also be met.

c. If it is necessary to check individual safety features during or after assembly, the method used shall be unambiguous and positive and shall not degrade safety.

13. **EXPLOSIVE ORDNANCE DISPOSAL (EOD)**

Features should be incorporated in fuzing systems in accordance with national policies that facilitate their being rendered safe by EOD tools, equipment and procedures even if de-arming, sterilisation or self-destruction features are incorporated.

DECLASSIFIED - PUBLICLY DISCLOSED - PDN(2016)0019 - DÉCLASSIFIÉ - MIS EN LECTURE PUBLIQUE

14. **DESIGN SAFETY ASSESSMENT**

a. Preliminary Safety Hazard Analysis. An analysis of the munition (including the fuze) shall be conducted to identify the hazards of normal and credible abnormal environments, conditions and actions of personnel, which may occur before the point of intended functioning is reached. This analysis shall be initiated at the start of a programme or in its early phases (STANAG 4297/AOP-15).

b. Integrated Design Analysis. The compliance of the fuzing system to the safety design requirements of this STANAG should be demonstrated with an integrated design analysis in accordance with the procedure laid out in AOP-42. This should be carried out in conjunction with the Hazard Analyses required in Paragraph 14c below.

c. Hazard Analyses. Analyses (Failure Modes and Effects Analysis, Fault Tree Analysis, etc.) shall be conducted and documented as soon as detailed design information is available. These analyses evaluate the safety of the fuzing system design in order to estimate the probabilities of a single system failing over its anticipated life cycle, including those due to manual operations. These probabilities shall not exceed the following rates:

(1) Prior to Commencement of the Arming Sequence. The probability of arming, or functioning irrespective of arming, between manufacture and the intended commencement of the arming sequence shall not exceed one in a million.

(2) Between Commencement of the Arming Sequence and Safe Separation or Equivalent Delay. The probability of arming between the intended commencement of the arming sequence and safe separation or equivalent delay shall not exceed one in a thousand. The rate of fuze functioning during this period shall be as low as is practical and consistent with the risk established as acceptable for premature munition function.

(3) After Safe Separation or Equivalent Delay. The probability of unintended functioning after safe separation or equivalent delay shall not exceed that specified in the requirement document for the system.

(4) After Intended Function. The design of the fuzing system shall ensure that the incidence of hazardous duds shall be at a level acceptable to the User and the NSAA.

d. Munitions including sub-munitions. For carrier munitions which include sub-munitions, the following shall apply:

(1) Probabilities and conditions given at Paragraph 14c shall apply to the fuzing system of the carrier munition.

(2) Probabilities and conditions given at Paragraphs 14c.(1) and 14c.(4) shall apply to the fuzing system of the sub-munition. Probabilities and conditions given at Paragraphs 14c.(2) and 14c.(3) shall also apply to the fuzing system of the sub-munition unless this is provided for in the carrier munition fuze.

The design authority shall demonstrate how the probability of unintended arming or functioning of a single sub-munition has been considered together with the total number of sub munitions to achieve a cumulative probability for the whole munition, that is acceptable to the NSAA.

The system hazard analysis shall demonstrate the requirements of Paragraph 6d.(3).

e. Hazard Analysis Revision. Hazard analyses shall be updated during development to assess the effects on safety of changes in the design.

f. Critical Components and Characteristics. Components of the fuzing system with characteristics that may be critical to safety shall be identified and appraised in a document that forms part of the fuzing system specification. The evaluation of the critical components shall be presented as part of the design safety assessment of the fuzing system. The applicable drawings shall be annotated to show that the component is safety critical together with the critical characteristics.

g. Embedded Software. For fuzing systems containing an embedded microprocessor, controller or other computing device, the analyses shall include a determination of the contribution of the software to the enabling of a safety feature. Where the software is shown to control directly or remove one or more of the safety features, a detailed analysis and testing of the applicable software shall be performed to assure that no design weaknesses, credible software failures, or credible hardware failures propagating through the software can result in a compromise of the safety features.

h. Safety Evaluation Documentation. The evaluation programme used as the basis of the safety assessment prepared by the design authority shall be documented in both detail and summary form. The Hazard Analysis relating to fuze failure probability, any revision and any analysis of Safety Critical Computing Systems shall be presented to the NSAA for assessment.

15. **DESIGN REVIEW**

Designs shall be certified by the NSAA for compliance with this STANAG. New designs, modifications to approved designs which affect safety, and new applications of previously approved designs shall be presented with supporting evidence to the NSAA for safety evaluation and certification of compliance.

16. **WAIVED REQUIREMENTS**

If a design does not comply with one or more requirements of this STANAG but is certified as safe by the NSAA, the details of the waived requirements and the rationale on which the waivers are based shall be recorded in the certificate of compliance. The reasons for the waivers shall be made known to other NATO nations justifiably requiring information on the design concerned.

17. **DESIGN FOR QUALITY CONTROL AND INSPECTION**

Fuzing systems shall be designed and documented to facilitate the application of effective quality control and inspection and test procedures in accordance with AQAP-110. The design of the fuzing system shall incorporate features that will facilitate the use of inspection procedures and test equipment to ensure that no critical design characteristics have been compromised.

IMPLEMENTATION OF THE AGREEMENT

18. The STANAG is implemented when NATO Members have issued instructions that all fuzing systems for all munition systems, except those listed in Paragraph 2.b., are to be designed in accordance with this agreement.

DECLASSIFIED - PUBLICLY DISCLOSED - PDN(2016)0019 - DÉCLASSIFIÉ - MIS EN LECTURE PUBLIQUE

NATIONAL SAFETY APPROVING AUTHORITIES

AUTORITES NATIONALES COMPETENTES EN MATIERE DE SECURITE

DECLASSIFIED - PUBLICLY DISCLOSED - PDN(2016)0019 - DÉCLASSIFIÉ - MIS EN LECTURE PUBLIQUE

CA Director General Ammunition,
National Defence Headquarters
101 Colonel By Drive
OTTAWA K1A0K2

DA Army Material Command,
Arsenalvej 55
DK-9800 HJORRING
Denmark

Navy Material Command,
Holmen
DK-1433 COPENHAGEN
Denmark

Air Material Command,
Postboks 130
DK-3500 VAERLOSE
Denmark

FR Etablissement Technique de Bourges
Centre Technique
Carrefour de O-NORD, Route de Guerry
BP 712
18015 BOURGES

Inspecteur de l'armement pour les poudres et explosifs
Cellule Sécurité des munitions (INSP/IPE/SM)
8 Boulevard Victor
00303 ARMEES

Laboratoire de Recherches Ballistiques et Aerodynamiques
Foret de Vernon
BP 914
27207 VERNON

GE Bundesamt fur Wehrtechnik und Beschaffung
WF I 5
Postfach 7360
D- 56057 KOBLENZ

IT Ministero della Difesa
Direzione Generale Armamenti Terrestri
Via Marsala n. 104
00185 ROMA

Marina Militaire (Navy)
Mariperman
Viale San Bartolomeo nr.396
19138 LA SPEZIA

DECLASSIFIED - PUBLICLY DISCLOSED - PDN(2016)0019 - DÉCLASSIFIÉ - MIS EN LECTURE PUBLIQUE

NL Royal Naval Material Command (DMkM)
Frederik Kazerne
PO Box 20702
2500 ES Den Haag

Royal Army Material Command (DMkL)
Ammunition Division
Frederik Kazerne
PO Box 90822
2509 LV Den Haag

Royal Air Force Material Command (DMkLU)
Brinkhorsthof
PO Box 20703
2500 ES Den Haag

NO Norwegian Army Material Command
Weapon and Ammunition Branch/ROMAN 6.2
OSLO Mil/Loren
0018 OSLO 1

UK Defence Ordnance Safety Group
Land Manoeuvre Division
Ash 2c
MOD Abbey Wood #3218
PO Box 702
BRISTOL BS12 7DU

US Army
Chairman,
US Army Fuze Safety Review Board
Attn: AMSTA-AR-CCZ
PICATINNY ARSENAL, NJ 07806-5000

Navy and Marine Corps

Chairman
Weapon System Explosives Safety Review Board
Naval Ordnance Safety & Security Activity
23 Strauss Ave., Building D-323
Indian Head, MD 20640-5070

Air Force

USAF
Non-nuclear Munitions Safety Board
Attn: AAC/SES
1001 North 2nd Street, Suite 366
Eglin Air Force Base
FL 32542-6838

DECLASSIFIED - PUBLICLY DISCLOSED - PDN(2016)0019 - DÉCLASSIFIÉ - MIS EN LECTURE PUBLIQUE

DEFINITIONS

1. For the interpretation of STANAG 4187 and AOP-16 the following specific definitions apply in addition to those in AAP-6. Definitions relating to the possible states that a fuzing system may adopt are given at Appendix 1 to this Annex

a. Armed. See Appendix 1, Table 3

b. Arming Delay. A delay between initiation of commit-to-arm, launch or deployment and arming of the fuzing system.

c. Commit-to-Arm. Actions carried out upon a munition, following which the fuzing system, irreversibly, will arm.

d. Common Cause Failure. The failure of two or more components due to a single cause. For example two or more components may fail due to the single cause of heating. The mode of failure may or may not be the same.

e. Common Mode Failure. The failure of two or more components in the same mode. For example two or more components such as switches may fail in a single mode such as open circuit. The cause of failure may or may not be the same.

f. Deactivated. See Appendix 1, Table 2.

g. De-Armed. See Appendix 1, Table 3.

h. Embedded Software. Software residing in the computer in 'Read Only' memory.

i. Enable. To remove or deactivate safety features which prevent arming.

j. Energy Break. A component, e.g., a switch, which prevents the accumulation of arming energy on the firing capacitor in a non-interrupted explosive train.

k. Environmental Sensor. A component or series of components designed to detect and respond to a specific environment.

l. Fail-Safe. A design feature of a fuzing system which renders the munition incapable of arming and functioning upon malfunction of safety feature(s) or exposure to out of sequence arming stimuli or operation of components.

m. Firing Stimulus. A stimulus which will initiate the first explosive element in the explosive train of a fuzing system.

n. Fuzing System. A system designed to:

(1) Provide as a primary role safety and arming functions in order to preclude munition arming before the desired position or time.

(2) Sense a target or respond to one or more prescribed conditions, such as elapsed time, pressure, or command.

(3) Initiate a train of fire or detonation in a munition.

o. Hand Emplaced Munition (HEM). A munition that is manually emplaced at, or is hand thrown to, a point of intended function and that requires user action both to begin its operation and to achieve safe separation. Examples include some demolition charges, grenades and pyrotechnics.

DECLASSIFIED - PUBLICLY DISCLOSED - PDN(2016)0019 - DÉCLASSIFIÉ - MIS EN LECTURE PUBLIQUE

p. Incendiary Mixes. Incendiary mixes are pyrotechnic compositions, which upon ignition rapidly convert to high temperature gases and hot particles.

q. Independent Safety Feature. A safety feature which is not affected by the function or malfunction of any other safety feature.

r. Interrupted Explosive Train. An explosive train in which the explosive path between the primary explosive and the lead and booster explosives is functionally separated until arming.

s. Interrupter. A barrier which prevents the transmission of an explosive or burning effect between elements in an explosive train.

t. Launch. The intentional and irreversible discharging, firing, ejecting or releasing of a munition.

u. Launch Cycle. The period from the instant a munition is irreversibly committed to launch until it has left its launcher.

v. Logic Route. The mapping of all functional paths that can be taken through a system operation.

w. Maximum Non-Initiation Threshold (MNIT) Stimulus For Arming. The energy stimulus at which the probability of functioning the initiator is. 0.005 at the 95% single sided lower level of confidence. Stimulus refers to the characteristic(s) such as current, rate of change of current (di/dt), power, voltage, or energy which is (are) most critical in defining the no-fire performance of the initiator.

x. No-Fire Threshold (NFT) Stimulus. The energy stimulus at which the probability of functioning the initiator is 0.001 at the 95% single sided lower level of confidence.

y. Non-Interrupted Explosive Train. An explosive train which has no physical interruption of the explosive elements.

z. Primary Explosives. Sensitive materials used to initiate a detonation or burning reaction.

aa Safe Jettison. Deliberate release or ejection of a non-armed munition in a manner which ensures that arming cannot occur.

bb. Safe Separation Distance. A minimum distance between the delivery system or launcher and the munition beyond which the hazards to personnel and the delivery system resulting from functioning of the munition system are acceptable.

cc. Safety and Arming Device (SAD). A device that prevents the fuzing system from arming until an acceptable set of conditions has been achieved and subsequently effects arming and allows functioning of the payload.

dd. Safety Critical Computing System. A computing system containing at least one Safety Critical Function.

ee. Safety Feature. An element or combination of elements of a fuzing system which prevent unintended arming and functioning.

ff. Sensitiveness. A measure of the ease with which an explosive may be ignited or initiated by a prescribed stimulus (an inverse measure of the safety of an explosive against accidental initiation).

gg. Shall. Indicates a provision that is mandatory.

hh. Should. Indicates a provision that, although not mandatory, is highly desirable, and, if it cannot be met, then the reasons shall be stated.

ii. Software. The non-hardware elements of a system which include computer programming operating systems, programming languages, data bases and associated documentation.

jj. Sterilised. See Appendix 1, Table 3.

kk. Stored Energy. The capability of a component to deliver energy in addition to any external energy required to initiate its function. Examples of stored energy are springs under load, batteries, charged capacitors, compressed gas devices and explosive actuators.

DECLASSIFIED - PUBLICLY DISCLOSED - PDN(2016)0019 - DÉCLASSIFIÉ - MIS EN LECTURE PUBLIQUE

DECLASSIFIED - PUBLICLY DISCLOSED - PDN(2016)0019 - DÉCLASSIFIÉ - MIS EN LECTURE PUBLIQUE

SENSOR\FUZE TERMS AND DEFINITIONS

1. **INTRODUCTION**

a. It has proved necessary to clarify the wide spectrum of possible states that both a Target Sensor and a Safety and Arming Device (SAD) can adopt within a fuzing system. Table 1 below describes the situation regarding the status of both the sensor and either an interrupted or non-interrupted SAD with the labels assigned for the status of the Target Sensor and the SAD. These terms are then defined in Tables 2 and 3. This table was originally developed to describe the various states that an intelligent mine (one that could be turned "on" and "off" for safe passage) might be in. It was decided to include this as it describes one or more states that all fuzed munitions can be expected to be in during their life cycle and use, whether they contain target sensors or not.

b. It is emphasised that the terms and definitions apply to the states that can be adopted within all types of fuzing system. For most fuzing systems, not all states are either possible or relevant.

c. When referring to a sensor, “Off” means that the sensor cannot produce an output (e.g., no firing signal).

d. When referring to a sensor, “On” means that the sensor can produce an output (e.g., firing signal).

e. Where charging circuitry is working, the firing capacitor can be expected to have a charge less than the Maximum Non-Initiation Threshold (MNIT) for only a very short time.

f. Where a firing capacitor has a charge greater than the MNIT , even in the absence of a working charging circuitry, the Electronic Safety and Arming Device (ESAD) is still armed.

g. A de-armed Safety and Arming Device is one which is returned to an unarmed condition having previously been armed.

Table 1 - Identification of Terms:

Ser	Target Sensor	SAD State	Interrupted Safety and Arming Device	Non-Interrupted Safety and Arming Device				Term for system status	
				Power Supply (SAD only)	Static Switches	Dynamic Switch	Firing Capacitor	Sensor	Fuze
(a)	(b)	(c)	(d)	(e)	(f)	(g)	(h)	(i)	(j)
1	Off	Unarmed	Interrupter locked, by at least 2 independent locking devices, in the position designed to prevent initiation of the main charge by the detonator.	Off or not available	Open	Not Oscillating	Not Charged	Inactive	Unarmed
2	On	Unarmed	As Above	On or off	Open	Not Oscillating	Not Charged	Active	Unarmed
3	On	Partially Armed	Interrupter in the position designed to prevent initiation of the main charge by the detonator but not fully locked in place as in serial 1.	On<br>On	Closed<br>Closed	Not Oscillating<br>Oscillating	Not Charged<br>Charged < MNIT	Active	Partially Armed
4	On	Armed	Interrupter in the position designed to allow initiation of the main charge by the detonator.	On	Closed	Oscillating	Charged > MNIT	Active	Armed
5	Functioned	Fired	Fired	Fired				Functioned	Functioned
6	Off	Armed	Interrupter in the position designed to allow initiation of main charge ready to fire.	On<br>Off	Closed<br>Closed or open	Oscillating<br>Not oscillating	Charged > MNIT<br>Charged > MNIT	De-Activated	Armed
7	Off	Partially De-Armed	Interrupter returned or progressed to a position designed to prevent initiation of the main charge but not fully locked in place. The SAD can be rearmed.	On or Off<br>On or Off	Closed<br>Open	Not Oscillating<br>Not Oscillating	Charged < MNIT<br>Charged < MNIT	De-Activated	Partially De-Armed
8	Off	De-Armed	Interrupter returned or progressed to an unarmed position and fully locked in place in such a manner that it can be rearmed.	Off	Open	Not Oscillating	Safely Discharged	De-Activated	De-Armed
9	Off	Sterilised	Interrupter moved from the armed position and returned to the position at which detonator function will not initiate the main charge and is permanently disabled. This may be achieved by functioning the detonator in that position.	Rendered permanently inoperable				Permanently De-Activated	Sterilised

Ser	Target Sensor	SAD State	Interrupted Safety and Arming Device	Non-Interrupted Safety and Arming Device				Term for system status	
				Power Supply (SAD only)	Static Switches	Dynamic Switch	Firing Capacitor	Sensor	Fuze
(a)	(b)	(c)	(d)	(e)	(f)	(g)	(h)	(i)	(j)
10	NA	Destroyed	SA mechanism armed and main charge functioned after a period of time or environmental condition has been sensed with the purpose of demolishing the munition and leaving no explosive hazard.	Fired and irreparably damaged				Destroyed	Self Functioned
11	NA	Destroyed	SA mechanism (or secondary SA mechanism) armed and subsidiary charge functioned after a period of time or environmental condition has been sensed with the purpose of disrupting the munition without functioning the main charge.	Disrupted	Disrupted	Disrupted	Fired	Destroyed	Self Disrupted

2. DEFINITION OF TERMS RELATED TO SENSORS

a. In the table below are the definitions related to the possible states that a Target Sensor for a fuzing system may adopt.

Table 2 - Definition of Terms Related to Sensors:

Ser	Term	Definition
(a)	(b)	(c)
1	Inactive	Munition sensor not turned on for the first time.
2	Active	Munition sensor turned on, capable of responding to a target and producing an output (e.g., firing signal).
3	Deactivated	Munition sensor turned off, having been turned on, and capable of being returned to the active state.
4	Permanently Deactivated	Munition sensor turned off, having been turned on, but incapable of being returned to the active state.
5	Destroyed	Munition sensor no longer assembled and incapable of being re-assembled and used.

3 DEFINITION OF TERMS RELATED TO SAFETY AND ARMING DEVICES

a. In the table below are the definitions related to the possible states that a SAD for a fuzing system may adopt.

b. **Safety and Arming Device (SAD)**. A SAD may be either an Interrupted Explosive Train SAD or a Non-Interrupted Explosive Train SAD

c. **Firing Capacitor Energy (FCE)**. The energy stored in the firing capacitor at any time intended to be applied to the initiator by closure of the firing switch. This energy is not to be confused with that which is stored on any other capacitor used to close a firing switch.

Table 3 - Definitions Related to Safety and Arming Devices

Ser	Term	Definition	
		Interrupted Explosive Train SAD	Non-Interrupted Explosive Train SAD
(a)	(b)	(c)	(d)
1	Unarmed	Interrupter locked, by all safety features, in the original position designed to prevent initiation of the main charge by the detonator.	Firing Capacitor Energy (FCE) shall not be present. All safety features in their original unpowered condition shall prevent accumulation of FCE (power supply to the SAD is off).
2	Partially Armed	The interrupter is in any position where the probability of initiation of the main charge by the detonator is less than 0.005 at the 95% single sided lower level of confidence, but with the safety features not fully applied as in the unarmed state.	FCE is greater than in the unarmed state and/or the Safety Features are not all fully applied. The FCE is less than the MNIT of the initiator.
3	Armed	The interrupter position is such that the probability of propagation of the explosive train is $\geq$ 0.005 at the 95% single sided lower level of confidence.	The FCE is greater than or equal to the MNIT of the initiator.
4	Partially De-Armed	A state in which the SAD, having been armed, is in any configuration where the probability of initiation of the main charge by the detonator is less than 0.005 at the 95% single sided lower level of confidence, but with the safety features not fully applied as in the de-armed state.	After having been armed, the FCE is greater than in the unarmed state and/or not all the Safety Features are fully applied. The FCE is less than the MNIT of the initiator.

Ser	Term	Definition	
		Interrupted Explosive Train SAD	Non-Interrupted Explosive Train SAD
(a)	(b)	(c)	(d)
5	De-Armed	A state in which the SAD, having been armed meets all of the following:<br>a. Is rendered incapable of functioning the main charge.<br>b. Meets the safety requirements of Paragraph 8.a.<br>c. Can be rearmed.	A state in which the SAD, having been armed meets all of the following:<br>a. Firing capacitor energy shall not be present.<br>b. Meets the safety requirement of Paragraph 8.b.<br>c. Can be rearmed.
6	Sterilised	A state in which the SAD is rendered permanently incapable of functioning the main charge. This shall be accomplished by either removal of the detonator or permanent interruption of the explosive train, or similar means.	A state in which the SAD is rendered permanently incapable of functioning the main charge.
7	Self functioned	The SAD is armed and functioned deliberately, without necessarily sensing a target, with the purpose of functioning the main charge.	The SAD is armed and functioned deliberately, without necessarily sensing a target, with the purpose of functioning the main charge.
8	Self disrupted	The SAD (or Secondary SAD) is functioned deliberately, without necessarily sensing a target, in order to operate a specific mechanism with the purpose of breaking up the munition without functioning the main charge.	The SAD (or Secondary SAD) is functioned deliberately, without necessarily sensing a target, in order to operate a specific mechanism with the purpose of breaking up the munition without functioning the main charge.

DECLASSIFIED - PUBLICLY DISCLOSED - PDN(2016)0019 - DÉCLASSIFIÉ - MIS EN LECTURE PUBLIQUE

ADDITIONAL SAFETY DESIGN REQUIREMENTS FOR MINE FUZING SYSTEMS

1. The design of safety and arming systems of all mine systems shall comply with the safety design requirements of this STANAG. There are additional requirements for mine fuzing or safety and arming systems, for example recovery and redeployment, and these are described in this Annex.

2. The mines referred to in this Annex may fire either a direct lethal mechanism or consist of a deployed launcher and sub-munition(s). The safety and arming mechanism in either the deployed launcher or the direct lethal mechanism is referred to as the SAD throughout this Annex. The SAD of a deployed launcher controls the firing of the expelling charge, whereas the SAD of a direct lethal mechanism controls the firing of the warhead. The SAD of any sub-munition shall be designed in accordance with the requirements of the main body of this STANAG.

3. Within this Annex, mine fuzing systems are divided into two functional parts:

a. The Target Sensor. The Target Sensor is a component or series of components designed to detect and respond to a target.

b. The Safety and Arming Device (SAD). A device that prevents the fuzing system from arming until an acceptable set of conditions has been achieved and subsequently effects arming and allows functioning of the payload.

4. Some mine systems also include a command, control and communications (C^3) subsystem. In such cases the C^3 sub-system shall be included in the munition design safety assessment (Paragraph 14 of the STANAG) to decide if any of its functions is safety critical, e.g., remote control of arming. The C^3 sub-system shall be able to validate the status of the mine at any stage of its operational deployment. If the assessment shows that the C^3 sub-system is safety critical, the design authority shall demonstrate that the requirements of this STANAG are not adversely affected.

5. Definitions. Those Definitions set out in the Tables 2 and 3 of Appendix 1 to Annex B are used to describe the states which may be adopted by the Target Sensor and the SAD.

6. Deployment. The target sensor should not be activated until the arming sequence of the SAD has been completed. Where this is not the case the design authority shall demonstrate to the NSAA how the safety requirements of Paragraph 14c.(2) of this STANAG are met.

7. Passage of friendly forces.

a. A system, designed to allow the passage of friendly forces, is recognised to be inherently less safe when set to this operational scenario. For this reason operational requirements shall justify such use and commanders shall be made aware of this hazard. Live munitions should not be used in this scenario during training. The design safety assessment shall demonstrate that the level of this hazard is acceptable to the User and the NSAA.

b. To allow the operational passage of friendly forces (operational passage mode):

(1) The SAD shall be in the de-armed state.

(2) The target sensor shall be deactivated.

(3) The firing circuit of a direct lethal mechanism or the launcher, in the case of a deployed launcher and sub-munition, shall be disabled.

(4) The remote command to re-arm shall require the operator to perform at least two different actions in a specific sequence, to generate and send a unique signal. If an external command is used to

initiate reactivation, the fuzing system shall validate the command before re-arming and shall not react to an invalid or corrupted command.

(5) Command and control of deactivation and activation of the target sensor shall be independent of the command and control of the SAD so that no common mode failure shall be able to effect the target sensor and the SAD. This shall be demonstrated to the NSAA.

(6) No failure of any part of the fuzing system related solely to re-arming may inhibit partial de-arming, de-arming, sterilisation, self-function or self-disrupt at a later time.

8. Approaching a Mine. If there is a User requirement to approach a mine, the design authority shall demonstrate how this could be achieved with the required safety.

9. Recovery. For a mine to be recovered, the fuzing system shall be at the unarmed or sterilised state with the target sensor deactivated.

10. Re-deployment. After being recovered, for a mine to be re-deployed the fuzing system must be in an unarmed state with the target sensor deactivated.

11. Self-Destruct. Self-destruction of a mine may be accomplished either by Self-Function or Self-Disrupt.

12. Where it is intended to use a mine fuzing system which incorporates a Non-Interrupted Explosive Train SAD, the accumulation of FCE shall be prevented until, and as late as possible, in the engagement sequence.

13. Fail-Safe. The failure of any component of the fuzing system which is not directly involved with de-arming, sterilisation, self-function or self-disrupt shall not compromise these capabilities.

14. End Of Deployed Life. Mines shall either self-destruct or auto-sterilise at the end of their planned usage, at the end of their laid life or on system malfunction. Self-disposal is intended to minimise the hazard of an unexploded mine. This function shall be included in the design safety assessment to ensure that the incidence of unexploded ordnance is at a level acceptable to the User and the NSAA.

DECLASSIFIED - PUBLICLY DISCLOSED - PDN(2016)0019 - DÉCLASSIFIÉ - MIS EN LECTURE PUBLIQUE

DECLASSIFIED - PUBLICLY DISCLOSED - PDN(2016)0019 - DÉCLASSIFIÉ - MIS EN LECTURE PUBLIQUE

PYROTECHNIC INITIATED EXPLOSIVE PROJECTILES

1. **GENERAL**

a. Pyrotechnic Initiated Explosive (PIE) projectiles utilise non-interrupted explosive trains and yet have no safety and arming systems in the accepted sense. It is therefore necessary to ensure that the explosives and other compositions used in the projectile are safe and suitable for service use in the storage, gun operation and in flight phases.

b. This Annex establishes the design safety requirements for PIE projectiles independently from the body of the STANAG.

c. The requirements of this Annex apply only to PIE projectiles of calibres of 40 mm and below.

2. **DEFINITIONS**

a. The definitions given in Annex B of this STANAG apply to the terms used herein.

3. **DESCRIPTION**

a. A PIE projectile is one in which:

(1) The explosive train is non-interrupted.

(2) There is no safety and arming device in the accepted sense and safety is achieved by the use of:

(a) Relatively insensitive incendiary mixes.

(b) Specific impulse levels to achieve initiation.

(c) A combination of projectile configuration and incendiary insensitivity.

(3) The explosive charge is ignited by the effects of combustion of incendiary mixes which in turn are ignited by impact on the target and by control of shock energy transfer through physical configuration.

4. **SAFETY DESIGN REQUIREMENTS**

a. Incendiary Mixes. The incendiary mixes:

(1) Shall be assessed and qualified in accordance with the principles and methodology described in STANAG 4170. Pass/fail criteria shall be established by the NSAA.

(2) Shall not contain material that could contribute to the subsequent formation of more volatile or more sensitive compounds.

(3) Shall not be altered by any means likely to increase their sensitiveness beyond that for which the materials were qualified.

b. The Explosive Charge. The explosive charge shall be assessed and Qualified in accordance with the principles and methodology described in STANAG 4170. Only those explosives that qualify as leads, booster or main charge explosives may be used as the explosive charge.

c. Materials. All of the materials within the projectile shall be chosen:

(1) So that the system is safe and remains so under all specified conditions of storage and use.

(2) To be compatible and stable so that under all specified natural and induced environmental conditions in its life cycle, none of the following can occur:

4.3.2.1. Premature functioning.

4.3.2.2. Dangerous ejection or exudation of material.

d. Round. The round:

(1) Shall be assessed in accordance with STANAG 4423.

(2) Shall be so designed that no credible circumstance can result in ignition of the explosive train before being fired.

(3) The round shall be tested in accordance with STANAG 4157 and subjected to the following additional tests. Specific test procedures and pass/fail criteria shall be established or approved by the NSAA:

(a) Double Ram Feed Test.

(b) Sympathetic Detonation.

(c) Brush Sensitivity.

(d) 12m Drop (with bare round and onto the nose)

(e) Impact Shock.

(f) Explosive Decompression.

(g) Acoustic Shock.

(4) Shall be subjected to hazard analysis, the results of which are to be acceptable to the NSAA.

(5) Shall require the impact energy that causes ignition of the explosive train to be at levels sufficiently high to be acceptable to the NSAA.

DECLASSIFIED - PUBLICLY DISCLOSED - PDN(2016)0019 - DÉCLASSIFIÉ - MIS EN LECTURE PUBLIQUE
